# Exercise-Induced Hypertension Is Associated with Cardiac Remodeling in Middle-Aged Male Long-Distance Runners: A Cross-Sectional Study

**DOI:** 10.3390/life16050853

**Published:** 2026-05-21

**Authors:** Young-Joo Kim, Han-Soo Park, Sang-Hyun Nam, Si-Eun Lee, Kyung-Hee Lee, Yongbum Park, Jin-Ho Yoon, Mu-Yeop Ji

**Affiliations:** 1 School of Sports Science, Sungshin Women’s University, Seoul 02844, Republic of Korea; kyj87@sungshin.ac.kr; 2 Department of Sports Medicine, Korean National Sport University, Seoul 05541, Republic of Korea; 302673@knsu.ac.kr; 3 Department of Plastic and Reconstructive Surgery, Sanggye Paik Hospital, Seoul 01757, Republic of Korea; s2607@paik.ac.kr; 4 Department of Physical Education, Sungshin Women’s University, Seoul 02844, Republic of Korea; sieun12053@hanmail.net; 5 Department of Exercise Therapy, Gachon University, Seoul 13120, Republic of Korea; cmalee2002@hanmail.net; 6 Department of Rehabilitation Medicine, Sanggye Paik Hospital, Inje University, Seoul 01757, Republic of Korea; swc328@naver.com; 7 Department of Sports Rehabilitation, Korea Nazarene University, Cheonan 31172, Republic of Korea

**Keywords:** exercise-induced hypertension, left ventricular hypertrophy, diastolic function, echocardiography, cardiac remodeling, endurance athletes, blood pressure response, middle-aged runners

## Abstract

Exercise-induced hypertension (EIH) has been linked to unfavorable cardiovascular outcomes; however, its implications for cardiac structure and function in middle-aged endurance athletes remain unclear. In the present study, cardiac remodeling was assessed using echocardiographic indicators, including left atrial diameter (LAD), left ventricular wall thickness, left ventricular mass (LVM), left ventricular mass index (LVMI), and the E/E′ ratio. This cross-sectional investigation examined 73 male long-distance runners aged 40–65 years, defined as individuals with ≥5 years of running experience and regular endurance training, classified according to maximal systolic blood pressure (SBPmax) during graded exercise testing: an exercise-induced hypertension group (EIHg; n = 35) and a non-EIH group (NEIHg; n = 38). Compared with the NEIHg, runners in the EIHg exhibited greater LAD, wall thickness, LVM, and LVMI (*p* < 0.05), whereas systolic function did not differ between groups. The E/E′ ratio was higher in the EIHg, suggesting subclinical alterations in diastolic function. LAD correlated with SBPmax, maximal diastolic blood pressure (DBPmax), LVM, and LVMI, while LVMI correlated with SBPmax but not resting systolic blood pressure. E/E′ was associated with DBPmax, LVM, and LVMI. Exploratory multivariable regression analysis showed that peak exercise systolic blood pressure remained associated with LVMI, whereas LAD was associated with SBPmax, BMI, and VO_2_max. These findings suggest that exaggerated exercise blood pressure responses are associated with cardiac remodeling and early diastolic alterations in middle-aged endurance runners. However, causal relationships cannot be established due to the cross-sectional design.

## 1. Introduction

Habitual physical activity improves cardiac function, supports vascular endothelial health, lowers the risk of cardiovascular disease, and decreases mortality [1]. Increased cardiac biomarkers have been reported after strenuous exercise, such as marathons and ultramarathons, and are thought to be related to sudden cardiac death, but the mechanisms underlying this increase remain unclear [2,3].

Sustained high-volume exercise training promotes arterial stiffening through endothelial dysfunction [4], leading to a higher incidence of coronary artery stenosis than in the general population [5]. Compared with normotensive runners, those with EIH demonstrate greater myocardial strain and a higher frequency of exercise-induced arrhythmias [6]. Additionally, they show a higher prevalence of coronary artery plaque [7]. In particular, higher cardiac biomarkers have been observed in individuals with EIH during or after long-distance events [8]. Such elevations reflect considerable myocardial stress and raise concerns regarding long-term health consequences [9]. In long-distance runners with EIH, prolonged excessive exercise may increase mechanical stress, arterial stiffness, and afterload, thereby imposing considerable strain on the myocardium [9,10]. Among the general population, EIH has been identified as a risk factor for cardiovascular and cerebrovascular diseases, sudden cardiac death, and premature mortality [11,12,13]. Recent studies indicate that middle-aged long-distance runners may face an increased risk of sudden cardiac death due to excessive myocardial strain related to EIH, potentially triggering fatal arrhythmias or coronary plaque rupture during physical activity [14]. Middle-aged runners with latent hypertension [15] and left ventricular hypertrophy (LVH) may have difficulty distinguishing pathological conditions from physiological adaptations to exercise, potentially leading to missed opportunities for accurate evaluation and timely treatment [16]. Although cardiac remodeling is a well-recognized adaptation in overtrained athletes, long-distance runners with hypertension during training may have difficulty distinguishing physiological adaptation from pathological change. Therefore, this study aimed to investigate cardiac structure and function in runners with EIH, using graded exercise testing (GXT), exercise habit data, and echocardiography. In this study, cardiac remodeling was defined on the basis of echocardiographic markers reflecting structural and functional cardiac adaptation, including LAD, LVPWd, IVSd, LVM, LVMI, and E/E′. In addition, we sought to identify factors associated with myocardial remodeling and explore the potential clinical implications of these findings.

## 2. Materials and Methods

### 2.1. Participants and Study Protocol

**Figure 1:**
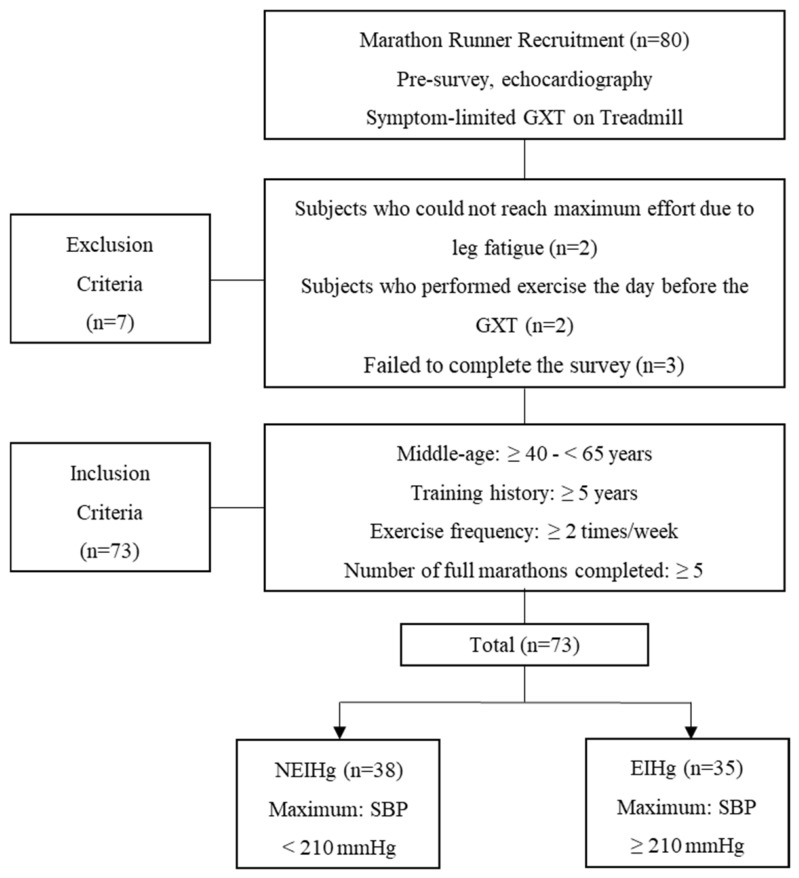
Flow chart of the study procedure. GXT: graded exercise testing; NEIHg: non-exercise-induced hypertension group; EIHg: exercise-induced hypertension group.

The cross-sectional study and participant flow are illustrated in Figure 1. This study was designed as a cross-sectional observational investigation to examine the association between exercise-induced hypertension (EIH) and echocardiographic markers of cardiac remodeling in middle-aged male long-distance runners. All participants underwent a symptom-limited graded exercise test (GXT) and resting echocardiographic evaluation according to a predefined study protocol.

In this study, long-distance runners were operationally defined as male runners aged 40–65 years with ≥5 years of running experience, regular endurance training ≥2 times per week, and completion of at least five full marathons. Participants were included if they met all of these criteria. Participants were excluded if they had known cardiovascular disease, musculoskeletal or neurological conditions limiting exercise performance, or any medical condition that could interfere with maximal exercise testing or echocardiographic assessment; these criteria were predefined but were not met by any participants.

Exercise-induced hypertension (EIH) was defined as a maximal systolic blood pressure (SBPmax) of ≥210 mmHg during maximal exercise testing in men [17]. Based on this criterion, participants were categorized into the non-exercise-induced hypertension group (NEIHg; n = 38) and the exercise-induced hypertension group (EIHg; n = 35). An a priori sample size calculation was conducted using G*Power software (version 3.1.9.7; Heinrich-Heine-Universität Düsseldorf, Düsseldorf, Germany). The calculation was based on a two-tailed independent *t*-test, an alpha level of 0.05, a statistical power of 0.80, and an assumed Cohen’s d of 0.80, corresponding to a large effect size. The minimum required sample size was lower than the final enrolled sample of 73 participants. This sample size calculation was primarily designed to ensure adequate statistical power for between-group comparisons.

### 2.2. Graded Exercise Test

Each participant underwent a symptom-limited GXT to assess hemodynamic responses and cardiorespiratory fitness across rest, exercise, peak exertion, and the recovery phase. Exercise testing variables included resting systolic and diastolic blood pressure (SBPrest and DBPrest), maximal systolic and diastolic blood pressure (SBPmax and DBPmax), maximal oxygen uptake (VO_2_max), metabolic equivalents (METs), and exercise duration. Blood pressure was measured at rest and during each stage of exercise using an automated sphygmomanometer, and VO_2_max was determined using a breath-by-breath metabolic analyzer. The GXT was conducted on a treadmill (h/p/cosmos, Nussdorf-Traunstein, Germany) using the Bruce protocol, during which blood pressure, heart rate, oxygen consumption, and electrocardiogram were measured. The equipment used included an electrocardiography system (CH2000, Cambridge Heart, Tewksbury, MA, USA), a breath-by-breath metabolic analyzer (Quark CPET, Cosmed, Rome, Italy), and an exercise blood pressure monitor (Tango+, Suntech, Morrisville, NC, USA). The Borg Scale was used to rate perceived exertion and gauge participants’ exercise intensity. Blood pressure was measured via a high-fidelity microphone applied directly over the brachial artery, with the examiner wearing headphones to ensure measurement accuracy. The entire GXT protocol adhered to recommendations issued by the American College of Cardiology and the American Heart Association [18].

### 2.3. Echocardiography Test

All echocardiographic examinations were performed by a single experienced and certified echocardiographer. The examination equipment was an Alpha 6 (Hitachi Aloka, Tokyo, Japan) system with a 5 MHz transducer. After resting for a sufficient period of 5 min, the heart rate and blood pressure were monitored. To assess the structure and function of the heart at rest, M-mode echocardiography was performed to record the following parameters: left atrial diameter (LAD), left ventricular internal dimension at end-diastole (LVIDd), left ventricular internal dimension at end-systole (LVIDs), left ventricular posterior wall thickness at end-diastole (LVPWd), interventricular septum thickness at end-diastole (IVSd), left ventricular mass (LVM), left ventricular mass index (LVMI), left ventricular end-diastolic volume (LVEDV), left ventricular end-diastolic stroke volume (LVESV), left ventricular stroke volume (LVSV), left ventricular stroke volume index (LVSVI), left ventricular ejection fraction (LVEF), cardiac output index (COI), left ventricular fractional shortening (LVFS), and cardiac output (CO). All measurements adhered to the standards of the American Society of Echocardiography (ASE), and all indices were indexed to body surface area. Diastolic function was evaluated using tissue Doppler imaging, which yielded the following parameters: peak velocity of early filling (E), peak velocity of atrial filling (A), early diastolic annulus velocity (E’), late diastolic annulus velocity (A’), deceleration time (DT), E/A ratio, E’/A’ ratio, and E/E’ ratio. Among these parameters, cardiac remodeling was operationally defined using LAD, LVPWd, IVSd, LVM, LVMI, and E/E′, which reflect structural remodeling and early diastolic alterations.

### 2.4. Statistical Analysis

Data are expressed as means ± standard deviations (Mean ± SD). All statistical procedures were carried out using SPSS Statistics version 21 (IBM Corporation, Armonk, NY, USA). Before applying inferential tests, distributional normality was verified with the Shapiro–Wilk test, and variance homogeneity was confirmed via Levene’s test to validate the use of parametric methods. For between-group comparisons, independent *t*-tests were conducted to determine significant differences in continuous variables, assuming that normality and homogeneity of variance were satisfied.

Associations between continuous variables were quantified using Pearson’s correlation coefficient (*r*). The strength of correlation was interpreted based on Cohen’s guidelines (small: 0.1–0.3; moderate: 0.3–0.5; large: >0.5). To further examine the independent association between exercise blood pressure and cardiac structural parameters, multivariable linear regression analyses were performed. LVMI and LAD were entered as dependent variables, while maximal systolic blood pressure during exercise (SBPmax) and potential confounders (age, BMI, resting systolic blood pressure, exercise history, exercise intensity, and VO_2_max) were included as independent variables. Effect sizes (standardized *β* coefficients) and 95% confidence intervals were reported for all regression analyses. A significance level of *α* = 0.05 was set for all statistical tests.

## 3. Results

### 3.1. Participant Characteristics

The general characteristics, hemodynamic parameters, and aerobic exercise capacity of the participants are presented in Table 1 (all comparisons indicate NEIHg vs. EIHg). No significant differences were observed between the groups in age, height, weight, BMI, smoking status, alcohol consumption, hypertension, hyperlipidemia, or diabetes mellitus. Regarding hemodynamic parameters, resting heart rate (HR), diastolic blood pressure (DBP), and maximum HR were similar between the groups. However, the resting systolic blood pressure (SBPrest) (121.7 ± 11.2 vs. 129.4 ± 13.2; *p* < 0.05), SBPmax (186.1 ± 13.0 vs. 224.1 ± 12.5; *p* < 0.05), and maximum DBP (DBPmax) (88.4 ± 7.1 vs. 96.3 ± 8.1; *p* < 0.05) were significantly higher in the EIHg compared with the NEIHg. During the recovery period following the GXT, SBP was measured every minute for three minutes. The results indicated that blood pressure levels were significantly higher in the EIHg than in the NEIHg during recovery, with recovery SBP being higher at one minute (183.5 ± 14.3 vs. 218.1 ± 14.4; *p* < 0.05), two minutes (174.5 ± 14.3 vs. 203.4 ± 15.3; *p* < 0.05), and three minutes (163.6 ± 14.2 vs. 189.3 ± 13.7; *p* < 0.05). No between-group difference was found in aerobic capacity, as indexed by VO_2_max.

### 3.2. Exercise-Related Variables

Table 2 presents the exercise-related characteristics of the participants. The two groups had no significant differences regarding training history, exercise time, exercise frequency, marathon time, or marathon completion. However, the exercise intensity (13.5 ± 1.4 vs. 14.3 ± 1.3, *p* < 0.05) was significantly higher in the EIHg than in the NEIHg.

### 3.3. Echocardiographic Findings

Table 3 presents echocardiographic comparisons between the NEIHg and EIHg (all values are presented as NEIHg vs. EIHg). There were no significant differences between the groups in terms of left ventricular structure and systolic function, as indicated by LVIDd (4.9 ± 0.4 vs. 5.0 ± 0.4) and LVIDs (3.2 ± 0.3 vs. 3.2 ± 0.3). LAD (3.5 ± 0.3 vs. 3.8 ± 0.3; *p* < 0.05), LVPWd (0.90 ± 0.09 vs. 0.96 ± 0.11; *p* < 0.05), IVSd (0.89 ± 0.08 vs. 0.99 ± 0.11; *p* < 0.05), LVM (179.5 ± 24.6 vs. 210.9 ± 35.3; *p* < 0.05), and LVMI (102.6 ± 13.0 vs. 117.9 ± 19.9; *p* < 0.05) were significantly higher in the EIHg than in the NEIHg. There were no significant differences between the groups in LVEDV (114.5 ± 20.8 vs. 116.8 ± 28.0), LVESV (40.3 ± 9.3 vs. 42.7 ± 10.2), LVSV (74.0 ± 13.8 vs. 76.7 ± 13.6), LVSVI (42.2 ± 6.8 vs. 42.9 ± 7.6), LVEF (64.9 ± 4.0 vs. 64.3 ± 4.5), CO (3.8 ± 0.7 vs. 4.3 ± 1.9), COI (2.1 ± 0.4 vs. 2.5 ± 1.0), or LVFS (36.5 ± 6.1 vs. 35.3 ± 3.3).

In terms of left ventricular diastolic function, both E-velocity (62.0 ± 15.3 vs. 70.0 ± 13.5; *p* < 0.05) and A-velocity (54.8 ± 10.8 vs. 63.5 ± 14.1; *p* < 0.05) were significantly higher in the EIHg compared with the NEIHg. E’-velocity (9.0 ± 1.8 vs. 9.0 ± 2.1), A’-velocity (10.7 ± 1.7 vs. 10.9 ± 1.4), DT (187.7 ± 21.2 vs. 184.1 ± 23.7), E/A ratio (1.18 ± 0.4 vs. 1.17 ± 0.4), and E’/A’ ratio (0.86 ± 0.2 vs. 0.83 ± 0.2) showed no significant differences between the groups. However, the E/E’ ratio (6.9 ± 1.8 vs. 8.0 ± 1.9, *p* < 0.05) was significantly higher in the EIHg than in the NEIHg.

### 3.4. Correlation Analysis

Table 4 presents the Pearson correlation coefficients of LAD and E/E’ with SBPrest, resting DBP (DBPrest), SBPmax, DBPmax, LVM, and LVMI. LAD showed no significant correlation with SBPrest (*r* = 0.15) or DBPrest (*r* = 0.11) but demonstrated significant correlations with SBPmax (*r* = 0.31), DBPmax (*r* = 0.23), LVM (*r* = 0.50), and LVMI (*r* = 0.49) (*p* < 0.05). E/E’ did not show significant correlations with SBPrest (*r* = 0.15), DBPrest (*r* = 0.07), or SBPmax (*r* = 0.10) but exhibited significant correlations with DBPmax (*r* = 0.32), LVM (*r* = 0.34), and LVMI (*r* = 0.33) (*p* < 0.05).

**Figure 2:**
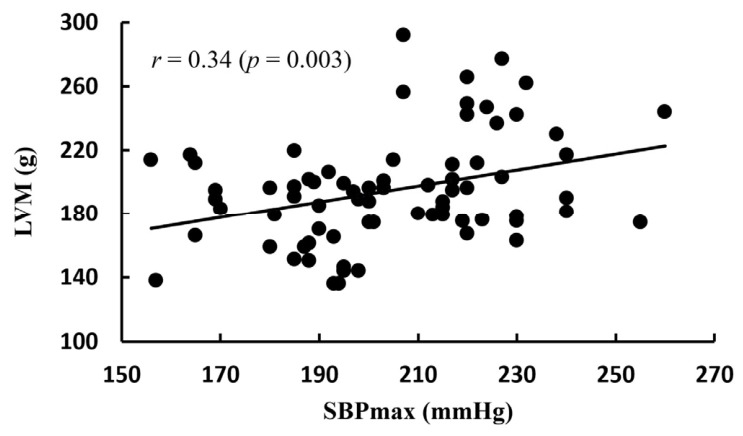
Correlation between LVM and SBPmax. LVM: left ventricular mass; SBPmax: maximum systolic blood pressure.

**Figure 3:**
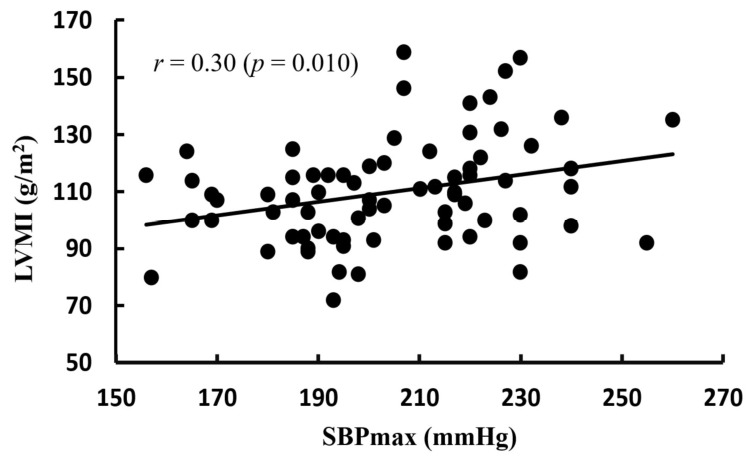
Correlation between LVMI and SBPmax. LVMI: left ventricular mass index; SBPmax: maximum systolic blood pressure.

**Figure 4:**
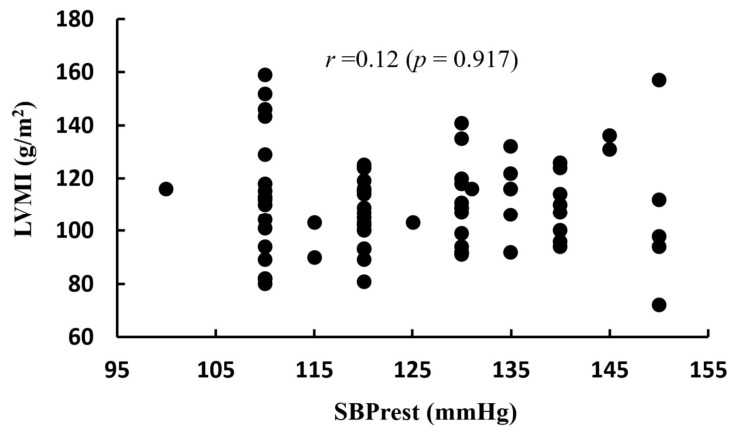
Correlation between LVMI and SBPrest. LVMI: left ventricular mass index; SBPrest: resting systolic blood pressure.

Figure 2 shows the correlation between LVM and SBPmax, Figure 3 presents the correlation between LVMI and SBPmax, and Figure 4 illustrates the correlation between LVMI and SBPrest. There was no significant correlation between LVMI and SBPrest, whereas a significant correlation was observed between LVMI and SBPmax.

### 3.5. Multivariable Regression Analysis

To further assess whether exercise blood pressure is independently associated with cardiac remodeling, multivariable linear regression analyses were conducted. In exploratory multivariable regression analyses, maximal systolic blood pressure during exercise (SBPmax) remained associated with LVMI (β = 0.369; *p* = 0.008; 95% CI: 0.078–0.509) after adjustment for age, BMI, resting systolic blood pressure, exercise history, exercise intensity, and VO_2_max (Table 5). In addition, SBPmax (β = 0.298; *p* = 0.022; 95% CI: 0.001–0.009), BMI (β = 0.405; *p* = 0.001; 95% CI: 0.036–0.142), and VO_2_max (β = 0.238; *p* = 0.045; 95% CI: 0.000–0.025) remained associated with LAD in the adjusted exploratory model (Table 5).

## 4. Discussion

The present study examined cardiovascular structure, function, and the clinical relevance of EIH in long-distance runners using echocardiographic assessment. Runners with EIH exhibited elevated blood pressure during maximal exertion, at rest, and during the recovery phase (Table 1). Strenuous exercise inherently elevates blood pressure, meaning that runners with EIH are subjected to substantially greater hemodynamic loads during physical activity. If these conditions persist chronically, it may be difficult to determine whether the observed myocardial remodeling is pathological. However, due to the cross-sectional design of this study, these findings should be interpreted as associations, rather than causal relationships.

The definition of EIH remains an important methodological issue, particularly in highly trained endurance athletes. In the present study, EIH was defined using the widely applied fixed threshold of SBPmax ≥ 210 mmHg in men. However, maximal exercise blood pressure is influenced by cardiorespiratory fitness, achieved workload, exercise protocol, and measurement technique. A recent large-scale EXERTION study demonstrated that exercise systolic blood pressure expressed relative to fitness, particularly SBP/METPeak, was associated with fatal and non-fatal cardiovascular events, whereas exercise systolic blood pressure without consideration of fitness was not associated with cardiovascular events [19]. Although the EXERTION study was conducted in a clinical exercise stress testing population rather than endurance athletes, its findings support the need to consider fitness- or workload-indexed blood pressure indices when interpreting EIH in trained individuals. Therefore, although the SBPmax ≥ 210 mmHg criterion used in the present study is clinically established and widely used, future studies in endurance athletes should consider incorporating indices such as SBP/METPeak, ΔSBP/ΔMET, or stage-specific blood pressure responses to better distinguish physiological adaptation from potentially maladaptive exercise blood pressure responses.

It was noted that, among the various exercise-related background factors, only the EIHg exhibited a higher habitual exercise intensity (Table 2). Intense endurance exercise can elevate biomarkers of reactive oxygen species, oxidative stress, and the oxidation of low-density lipoprotein, all of which may accelerate atherosclerosis [20,21]. It is recommended that exercise intensity be adjusted as a potential intervention to mitigate cardiovascular risk if negative outcomes are observed in the EIHg in future studies.

Echocardiographic data in Table 3 revealed that LAD was significantly larger in the EIHg compared with the NEIHg. Left atrial (LA) dilation is a well-documented finding among endurance athletes [22]. However, the significant increase in LAD observed in this study is more likely associated with EIH [23]. In hypertensive conditions, LA dilation is associated with LVM irrespective of hypertrophy type, driven by elevated filling pressures [24]. Athletes who engage in excessive exercise face a risk of developing atrial fibrillation that is up to five times higher than that of the general population [25]. This phenomenon is attributed to repetitive atrial overexpansion, inflammation, and fibrosis [26], which can potentially lead to sudden cardiac death [27]. Although LAD enlargement in athletes is frequently attributed to high training volumes, evidence specifically linking greater LAD dilation to EIH in runners remains limited. In this study, although LAD enlargement remained within the normal range, it showed a positive correlation with SBPmax, DBPmax, and LVMI, suggesting that it may be associated with exaggerated blood pressure responses during exercise (Table 4). Therefore, relative atrial enlargement in runners with EIH may indicate a potential risk of future atrial arrhythmia. Similarly, SBPmax remained associated with LAD in the adjusted exploratory model, although LAD enlargement appeared to be influenced by multiple factors, including body composition and aerobic fitness.

In the EIHg, the left ventricular hypertrophy (LVH) parameters―LVPWd, LVM, and LVMI―were significantly higher than in the NEIHg. Notably, the mean LVMI in the EIHg was 117.9 ± 19.9 g/m^2^, slightly exceeding the conventional upper reference threshold of 115 g/m^2^. However, because endurance training itself can increase LV mass, this finding should be interpreted cautiously and may reflect a mixture of physiological athletic remodeling and potentially maladaptive remodeling. In the general population, echocardiographically detected LVH is linked to substantially elevated risks of hypertension-related cardiovascular morbidity and mortality [28,29]. This includes elevated risks of coronary artery disease, congestive heart failure, stroke, and sudden cardiac death [30].

In athletic populations, LVH generally represents a structural adaptation to sustained exercise demands and is regarded as physiological remodeling, provided hypertrophic cardiomyopathy has been excluded [31].

Nevertheless, the more pronounced LVH in the EIHg relative to the NEIHg cannot be attributed solely to exercise-induced physiological adaptation. Furthermore, as the SBPrest in the EIHg was also found to be higher than that in the NEIHg, there is a greater likelihood of underlying hypertension. Therefore, in the absence of ABPM, the present findings should be interpreted as reflecting an association between exaggerated exercise blood pressure responses and cardiac remodeling, rather than evidence that remodeling was attributable solely to EIH independent of overall blood pressure exposure. A key clinical concern is that athletes presenting with mild hypertension or LVH may be incorrectly assumed to exhibit only exercise-related adaptations, potentially resulting in delayed diagnosis [18,24]. As aging is an independent risk factor for cardiovascular disease, particularly when combined with excessive and chronic exercise in middle age, it can accelerate the progression of atherosclerosis [4]. Prior research has reported that approximately 38% of middle-aged athletes harbor undiagnosed hypertension [32]. In middle-aged endurance athletes with LVH, this condition is associated with hypertension, which requires more proactive treatment measures [15,33]. This concern may become more clinically relevant as these athletes age. A recent consensus document from the European Society of Hypertension Working Group on Hypertension and the Heart emphasized that hypertensive heart disease in older adults is closely related to cumulative blood pressure exposure and age-related cardiovascular changes, including increased arterial stiffness, left ventricular remodeling, diastolic abnormalities, atrial enlargement, and heart failure risk [34]. In this context, EIH in middle-aged endurance athletes may represent an early phenotype of exaggerated blood pressure load, particularly when accompanied by higher resting SBP and increased LVMI. Although the present cross-sectional study cannot determine whether EIH directly progresses to hypertensive heart disease, our findings suggest that middle-aged runners with EIH may require longitudinal monitoring as they age to clarify whether repeated exercise-related pressure overload contributes to later hypertensive cardiac remodeling. According to Kim et al. [7], middle-aged long-distance runners with EIH exhibited a higher prevalence of coronary artery plaques; however, the coronary artery plaques were not detected during GXT, prompting the recommendation for more detailed evaluations using cardiac CT. Additionally, runners with EIH showed increased arterial stiffness [35] and greater carotid intima-media thickness compared with runners with normal blood pressure responses during exercise [36]. In this study, LVMI showed a positive correlation with SBPmax, independently of SBPrest (Figure 3 and Figure 4). These findings suggest that excessive blood pressure elevation during exercise may be more closely associated with an increased risk of LVH than SBPrest. Multivariable regression analysis further showed that exercise systolic blood pressure remained associated with LVMI after adjustment for selected covariates; however, given the modest sample size, this finding should be interpreted cautiously and confirmed in larger cohorts.

E/E’ (normal range: <8) is commonly used as a key indicator of left ventricular diastolic function [24]. Although E/E′ values remained within the normal range, a relative increase was observed in the EIHg, with E/E’ being significantly higher than in the NEIHg (8.0 ± 1.9 vs. 6.9 ± 1.8). In patients with chronic hypertension, the E/E’ ratio is typically elevated [37]. This study found a significant positive correlation between E/E’ and both DBPmax and LVMI (Table 4). This suggests that increased exercise diastolic blood pressure and greater LVMI may be associated with early or subclinical alterations in myocardial diastolic properties, rather than overt diastolic dysfunction. However, these findings should not be interpreted as evidence of overt pathological remodeling and warrant longitudinal monitoring in endurance athletes. Further follow-up is necessary to determine whether these factors influence cardiovascular remodeling or contribute to clinical cardiovascular events. This study has several limitations:

First, we could not perform 24 h ambulatory blood pressure monitoring (ABPM), which is crucial for definitively diagnosing hypertension in both groups, thereby limiting our ability to fully assess the underlying pathological risk. In particular, because resting SBP was significantly higher in the EIHg than in the NEIHg, the possibility of masked hypertension, early sustained hypertension, or higher overall blood pressure exposure cannot be excluded. Therefore, it remains difficult to determine whether the observed cardiac remodeling was specifically associated with EIH or reflected a broader cumulative blood pressure burden. As a result, our findings may not fully reflect the pathological risk, and future studies should incorporate ABPM to better characterize blood pressure phenotypes in endurance athletes.

Second, the definition of exercise-induced hypertension (EIH) was based on a fixed threshold (SBP ≥ 210 mmHg), which, although widely used, remains debated and may not fully account for physiological adaptations in highly trained endurance athletes. In addition, the present study did not index exercise blood pressure to achieved workload or cardiorespiratory fitness. Recent evidence suggests that fitness-indexed indices, such as SBP/METPeak, may improve cardiovascular risk discrimination beyond absolute exercise SBP values. Therefore, future studies should examine whether workload- or fitness-adjusted exercise blood pressure indices provide better classification of clinically meaningful EIH in endurance athletes.

Third, the findings of this study cannot be generalized to female populations, as only male participants were included, and sex-specific differences in cardiovascular responses should be considered in future research.

Fourth, although some participants with pre-existing hypertension, diabetes, or hyperlipidemia were included, the potential influence of other undiagnosed conditions or lifestyle factors on the study outcomes cannot be ruled out.

Fifth, because of the cross-sectional nature of this study, causal relationships between exercise blood pressure and cardiac remodeling cannot be established. Longitudinal studies are required to confirm these findings.

Sixth, although the total sample size exceeded the minimum requirement estimated for the primary between-group comparison, the sample size was modest for multivariable regression analyses involving several covariates. Therefore, the adjusted models should be interpreted as exploratory, and the stability of these associations requires confirmation in larger cohorts including both male and female endurance athletes.

## 5. Conclusions

In conclusion, exaggerated exercise blood pressure responses in middle-aged long-distance runners with EIH were associated with cardiac remodeling, particularly LVMI and LAD. These associations were also observed in exploratory adjusted models; however, given the modest sample size, the multivariable findings should be interpreted cautiously and confirmed in larger longitudinal studies. Although these findings suggest a potential link with increased cardiovascular risk, they are more consistent with early alterations in diastolic function than with overt dysfunction, highlighting the need for longitudinal studies to further clarify these relationships.

## Figures and Tables

**Table 1:** Characteristics of participants: baseline demographics, hemodynamics, and cardiorespiratory fitness.

	NEIHg (n = 38)	EIHg (n = 35)
General characteristics		
Age (years)	57.8 ± 6.9	58.3 ± 6.3
Height (cm)	170.7 ± 5.9	171.3 ± 7.5
Weight (kg)	64.5 ± 5.7	66.3 ± 6.2
BMI (kg/m^2^)	22.1 ± 1.5	22.8 ± 1.8
Smoking status (%)	0 (0%)	0 (0%)
Alcohol (%)	33 (86.8%)	32 (91.4%)
Hypertension (%)	1 (2.6%)	3 (8.6%)
Hyperlipidemia (%)	1 (2.6%)	3 (8.6%)
DM (%)	3 (7.9%)	1 (2.9%)
Hemodynamic characteristics		
HRrest (BPM)	54.7 ± 7.2	56.5 ± 9.5
HRmax (BPM)	162.7 ± 13.3	162.0 ± 13.3
SBPrest (mmHg)	121.7 ± 11.2	129.4 ± 13.2 *
SBPmax (mmHg)	186.1 ± 13.0	224.1 ± 12.5 *
DBPrest (mmHg)	78.4 ± 7.0	81.3 ± 8.9
DBPmax (mmHg)	88.4 ± 7.1	96.3 ± 8.1 *
RSBP1 (mmHg)	183.5 ± 14.3	218.1 ± 14.4 *
RSBP2 (mmHg)	174.5 ± 14.3	203.4 ± 15.3 *
RSBP3 (mmHg)	163.6 ± 14.2	189.3 ± 13.7 *
Aerobic exercise capacity		
VO_2_max (mL/kg/min)	50.1 ± 6.8	47.8 ± 6.8

Values are presented as numbers (%) or means ± standard deviations. NEIHg: non-exercise-induced hypertension group; EIHg: exercise-induced hypertension group; BMI: body mass index; DM: diabetes mellitus; HR: heart rate; BPM: beat per minute; SBP: systolic blood pressure; DBP: diastolic blood pressure; RSBP1: recovery systolic blood pressure at one minute; RSBP2: recovery systolic blood pressure at two minutes; RSBP3: recovery systolic blood pressure at three minutes. * *p* < 0.05 from NEIHg.

**Table 2:** Exercise data.

	NEIHg (n = 38)	EIHg (n = 35)
Training history (yrs)	17.8 ± 7.3	16.4 ± 5.7
Exercise intensity (Borg’s RPE scale)	13.5 ± 1.4	14.3 ± 1.3 *
Exercise time (min/day)	88.9 ± 28.9	77.4 ± 27.5
Exercise frequency (times per week)	4.2 ± 1.3	3.9 ± 1.4
Marathon time (min)	220.6 ± 28.8	225.2 ± 30.4
Marathons completed (number)	86.6 ± 79.0	63.1 ± 70.8

Values are presented as means ± standard deviations. NEIHg: non-exercise-induced hypertension group; EIHg: exercise-induced hypertension group. * *p* < 0.05 from NEIHg.

**Table 3:** Echocardiographic parameters.

	NEIHg (n = 38)	EIHg (n = 35)
Left ventricular structure and systolic function		
LAD (mm)	3.5 ± 0.3	3.8 ± 0.3 *
LVIDd (mm)	4.9 ± 0.4	5.0 ± 0.4
LVIDs (mm)	3.2 ± 0.3	3.2 ± 0.3
LVPWd (mm)	0.90 ± 0.09	0.96 ± 0.11 *
IVSd (mm)	0.89 ± 0.08	0.99 ± 0.11 *
LVM (g)	179.5 ± 24.6	210.9 ± 35.3 *
LVMI (g/m^2^)	102.6 ± 13.0	117.9 ± 19.9 *
LVEDV (mL)	114.5 ± 20.8	116.8 ± 28.0
LVESV (mL)	40.3 ± 9.3	42.7 ± 10.2
LVSV (mL)	74.0 ± 13.8	76.7 ± 13.6
LVSVI (mL/m^2^)	42.2 ± 6.8	42.9 ± 7.6
LVEF (%)	64.9 ± 4.0	64.3 ± 4.5
CO (L/min)	3.8 ± 0.7	4.3 ± 1.9
COI (L/min/m^2^)	2.1 ± 0.4	2.5 ± 1.0
LVFS (%)	36.5 ± 6.1	35.3 ± 3.3
Left ventricular diastolic function		
E-velocity (cm/s)	62.0 ± 15.3	70.0 ± 13.5 *
A-velocity (cm/s)	54.8 ± 10.8	63.5 ± 14.1 *
E’-velocity (cm/s)	9.0 ± 1.8	9.0 ± 2.1
A’-velocity (cm/s)	10.7 ± 1.7	10.9 ± 1.4
DT (m/s)	187.7 ± 21.2	184.1 ± 23.7
E/A	1.18 ± 0.4	1.17 ± 0.4
E’/A’	0.86 ± 0.2	0.83 ± 0.2
E/E’	6.9 ± 1.8	8.0 ± 1.9 *

Values are presented as means ± standard deviations. NEIHg: non-exercise-induced hypertension group; EIHg: exercise-induced hypertension group; LVIDd: left ventricular internal dimension end-diastolic; LVIDs: left ventricular internal dimension end-systolic; LVPWd: left ventricular posterior wall thickness end-diastolic; IVSd: interventricular septum thickness end-diastolic; LAD: left atrial diameter; LVM: left ventricular mass; LVMI: left ventricular mass index; LVEDV: left ventricular end-diastolic volume; LVESV: left ventricular end-systolic volume; LVSV: left ventricular stroke volume; LVSVI: left ventricular stroke volume index; LVEF: left ventricular ejection fraction; CO: cardiac output; COI: cardiac output index; LVFS: left ventricular fractional shortening; E: peak velocity of early filling; A: peak velocity of atrial filling; E’: early diastolic annulus velocity; A’: late diastolic annulus velocity; DT: deceleration time. * *p* < 0.05 from NEIHg.

**Table 4:** Correlation of blood pressure and LVM(I) for LAD and E/E’.

	SBPrest (mmHg)	DBPrest (mmHg)	SBPmax (mmHg)	DBPmax (mmHg)	LVM (g)	LVMI (g/m^2^)
LAD (mm)	*r* = 0.15	*r* = 0.11	*r* = 0.31 *	*r* = 0.23 *	*r* = 0.50 *	*r* = 0.49 *
E/E’	*r* = 0.15	*r* = 0.07	*r* = 0.10	*r* = 0.32 *	*r* = 0.34 *	*r* = 0.33 *

Values are presented as means ± standard deviations. LAD: left atrial diameter; E: peak velocity of early filling; E’: early diastolic annulus velocity; SBPrest: resting systolic blood pressure; DBPrest: resting diastolic blood pressure; SBPmax: maximal systolic blood pressure; DBPmax: maximal diastolic blood pressure; LVM: left ventricular mass; LVMI: left ventricular mass index. * *p* < 0.05.

**Table 5:** Multivariable linear regression analysis for predictors of left ventricular mass index (LVMI) and left atrial diameter (LAD).

Variable	B	SE	*β*	95% CI	*p*-Value
**Left** **ventricular mass index**					
Age	0.489	0.346	0.177	−0.203–1.180	0.163
BMI	1.753	1.401	0.160	−1.045–4.551	0.215
SBPrest	−0.302	0.193	−0.211	−0.688–0.085	0.124
Exercise history	0.378	0.357	0.136	−0.335–1.090	0.294
Exercise intensity	0.460	1.610	0.036	−2.756–3.676	0.776
VO_2_max	0.333	0.330	0.125	−0.327–0.992	0.318
SBPmax	0.293	0.108	0.369	0.078–0.509	0.008
**Left atrial diameter**					
Age	0.010	0.007	0.178	−0.003–0.023	0.136
BMI	0.089	0.026	0.405	0.036–0.142	0.001
SBPrest	−0.003	0.004	−0.102	−0.010–0.004	0.426
Exercise history	0.003	0.007	0.061	−0.010–0.017	0.616
Exercise intensity	0.006	0.030	0.024	−0.055–0.067	0.841
VO_2_max	0.013	0.006	0.238	0.000–0.025	0.045
SBPmax	0.005	0.002	0.298	0.001–0.009	0.022

BMI, body mass index; SBP, systolic blood pressure; VO_2_max, maximal oxygen uptake; MSBP, maximal systolic blood pressure; B, unstandardized coefficient; SE, standard error; β, standardized coefficient; CI, confidence interval.

## Data Availability

The data presented in this study are available on request from the corresponding authors. The data are not publicly available due to privacy and ethical restrictions.

## Abbreviations

The following abbreviations are used in this manuscript:

ABPM	Ambulatory blood pressure monitoring
ASE	American Society of Echocardiography
BMI	Body mass index
CO	Cardiac output
COI	Cardiac output index
DBP	Diastolic blood pressure
DBPmax	Maximum diastolic blood pressure
DBPrest	Resting diastolic blood pressure
DT	Deceleration time
E	Peak velocity of early filling
E’	Early diastolic annulus velocity
E/A	Ratio of early to late ventricular filling velocities
E’/A’	Ratio of early to late diastolic annulus velocity
E/E’	Ratio of early transmitral flow velocity to early diastolic annulus velocity
EIH	Exercise-induced hypertension
EIHg	Exercise-induced hypertension group
GXT	Graded exercise test
HR	Heart rate
HRmax	Maximum heart rate
HRrest	Resting heart rate
IVSd	Interventricular septum thickness at end-diastole
LA	Left atrium
LAD	Left atrial diameter
LVEDV	Left ventricular end-diastolic volume
LVESV	Left ventricular end-systolic volume
LVEF	Left ventricular ejection fraction
LVFS	Left ventricular fractional shortening
LVH	Left ventricular hypertrophy
LVIDd	Left ventricular internal dimension at end-diastole
LVIDs	Left ventricular internal dimension at end-systole
LVM	Left ventricular mass
LVMI	Left ventricular mass index
LVSV	Left ventricular stroke volume
LVSVI	Left ventricular stroke volume index
NEIHg	Non-exercise-induced hypertension group
RSBP1	Recovery systolic blood pressure at 1 min
RSBP2	Recovery systolic blood pressure at 2 min
RSBP3	Recovery systolic blood pressure at 3 min
SBP	Systolic blood pressure
SBPmax	Maximum systolic blood pressure
SBPrest	Resting systolic blood pressure
